# Effect of Bioactive Peptides on Gut Microbiota and Their Relations to Human Health

**DOI:** 10.3390/foods13121853

**Published:** 2024-06-13

**Authors:** Tharuka Wijesekara, Edirisinghe Dewage Nalaka Sandun Abeyrathne, Dong Uk Ahn

**Affiliations:** 1 Department of Food Science and Agricultural Chemistry, Faculty of Agricultural and Environmental Sciences, McGill University, Sainte-Anne-de-Bellevue, QC H9X 3V9, Canada; muhandiramlage.wijesekara@mail.mcgill.ca; 2 Department of Animal Science, Uva Wellassa University, Badulla 90000, Sri Lanka; 3 Department of Animal Science, Iowa State University, Ames, IA 50011, USA

**Keywords:** bioactive peptides, mechanisms of function, gut microbiota, gut health, human health

## Abstract

Bioactive peptides derived from both exogenous and endogenous origins have been studied extensively to use their beneficial effects in humans and animals. Bioactive peptides exhibit beneficial bodily functions and contribute to a healthy gastrointestinal system by influencing barrier functions, immune responses, and gut microbiota. Gut microbiota is a diverse microbial community that significantly influences the overall well-being and homeostasis of the body. Factors such as diet, age, lifestyle, medication, and environmental circumstances can affect the composition and diversity of the gut microbiota. The disturbances or imbalances in the gut microbiota have been associated with various health problems. The interplays between bioactive peptides and gut microbiota are not fully understood, but bioactive peptides hold promise as modulators of the gut microbiota to promote gut health. Almost all the bioactive research on human health, including the development of therapeutics and nutritional interventions, uses cell culture, even though their direct biofunctional activities can only occur when absorbed in the intestine and into the blood system. This review focuses on the current understanding of bioactive peptides in gut microbiota and their impact and mechanisms on gut and human health. The novelty of this review lies in its comprehensive analysis of the multifaceted interactions between bioactive peptides and gut microbiota, integrating knowledge from diverse disciplines between microbiology and nutrition. By elucidating the underlying mechanisms and identifying current research gaps, this review offers an outlook on the potential of bioactive peptides in promoting gut health and shaping future therapeutic and nutritional interventions.

## 1. Introduction

Gut microbiota harbors in the gastrointestinal tract of living organisms [1]. Although bacteria are the main group of this gut microbiota family, other microbes such as fungi, viruses, archaea, and protozoans also coexist within the alimentary tract [2]. Gut microbiota significantly influences the host’s physiological functions, especially in maintaining homeostasis and promoting overall well-being [3]. Gut microbiota exerts multifaceted roles, including nutrient metabolism, biosynthesis of vital molecules, modulation of the immune system, and defense against pathogens and xenobiotics, and its dysregulation may contribute to the pathogenesis of hosts under diverse conditions, including obesity, diabetes, inflammatory bowel disease, and mental disorders. Various factors, including diet, age, genetic makeup, lifestyle, medication, and environmental circumstances, can impact the composition and heterogeneity of gut microbiota [2,4].

Various bioactive peptides are recommended for their applications in the food, pharmaceutical, and cosmeceutical industries [4,5]. These bioactive peptides are derived from plants, animals, insects, fungi, and microbes [6,7]. They are reported to prevent chronic ailments, regulate physiological functions, impact gut microbiota, promote beneficial bacteria, and regulate immune function [8,9]. The interplays between bioactive peptides and gut microbiota are complex [9] and still not extensively studied [10]. Certain peptides influence the gut microbiota by enhancing beneficial bacteria and modulating immune responses [7]. They have diverse health-promoting effects and potential applications in therapeutics and nutritional interventions, particularly through the gut microbiota [11]. Endogenous bioactive peptides benefit health and are categorized into three types: produced through biosynthesis, directly encoded, and derived from cryptic proteins [12]. There is an increasing interest in exploring the development of food additives and functional products and the development of new therapeutics using these bioactive peptides [13]. This review investigates the effects of bioactive peptides on the gut microbiota and their potential applications in developing new therapeutics and nutritional interventions.

This review presents a comprehensive examination of the interactions between bioactive peptides and gut microbiota, highlighting their potential applications in promoting gut health and overall well-being. It uniquely integrates knowledge of how bioactive peptides influence gut microbiota and human health. By delving into the underlying mechanisms of peptide–microbiota interactions, the review provides deeper insights into their functional roles and identifies current research gaps, paving the way for future studies. The implications of these findings are significant, suggesting that bioactive peptides could be harnessed to develop new therapeutic and nutritional interventions aimed at preventing and managing chronic diseases linked to gut microbiota dysregulation.

## 2. Bioactive Peptides

### 2.1. Animal-Derived Bioactive Peptides

Animal-derived bioactive peptides are mostly derived from milk, meat, eggs, and fish [14]. However, not all peptides released from the original protein sources have bioactive properties. The animal breeds, the specific cut, and the cooking technique significantly affect the levels of all bioactive compounds [15].

Peptides derived from milk have various functions, including antimicrobial, immune-modulatory, antioxidant, enzyme-inhibitory, antithrombotic, and antagonistic effects against harmful agents [16]. Many phosphopeptides released from casein (CPPs) have been widely applied as mineral supplements and functional foods enriched with calcium or iron. Especially complexing CPPs with amorphous calcium phosphate (ACP) stabilized calcium and phosphate [17]. This CPP-ACP complex helped to re-mineralize enamel, reduced the streptococci mutan colonization and biofilm formation, and improved teeth health [18]. The phosphopeptides produced from egg yolk phosvitin are also expected to have similar functions and bioactivity to CPPs because they share common structural characteristics [19]. Requena et al. [20] found that consuming 750 mg/kg of egg white hydrolysate (EWH) for 12 weeks increases the amount of *Lactobacillus*/Enterococcus and *Clostridium leptum* bacteria in the gut and modifies the production of gut microbiota metabolites, notably elevating total short-chain fatty acids in feces. Fish protein hydrolysates and specific peptides derived from other hydrolysates have a wide range of biological activities [21]. Fish is a rich source of antimicrobial peptides, including defensins, cathelicidins, hepcidins, and peptides derived from histones [22]. Fishbone and other body parts such as gills and mucous layers are also identified as possible peptide sources [23].

An expanding body of research demonstrates the discovery of an ever-increasing number of insect-derived biopeptides. Recent studies involving in vitro and in vivo screening, utilizing surrogate model hosts like C. elegans, have highlighted the promising antimicrobial activity of insect-derived AMPs against human pathogens [24]. Mylonakis et al. [24] found that the insect cricket produces a peptide called Defensin-1, which exhibits antimicrobial properties. Another investigation focused on the silkworm and identified three peptides, namely SPKFCW, DQDPFRP, and PDPSKF, which also possess antimicrobial activity. These findings highlight the potential of insects as a source of bioactive peptides with antimicrobial properties, suggesting their possible applications in various fields such as medicine and biotechnology [25]. Table 1 shows the bioactivity of animal-derived peptides from various animal sources.

### 2.2. Plant-Derived Bioactive Peptides

Plant peptides can be produced from leaves, seeds, and fruits, with seeds being the most cost-effective protein source [36]. Soy proteins are a significant source of plant-derived bioactive peptides, with glycinin and beta-conglycinin accounting for more than 85% of soy proteins [37]. Plant-derived peptides have antimicrobial properties that inhibit the growth of *Escherichia coli*, *Staphylococcus aureus*, *Pseudomonas aeruginosa*, and *Salmonella enterica*. They also act as antioxidants by scavenging ABTS^+^ and DPPH radicals and inhibiting β-carotene oxidation and possess anticancer properties by inhibiting the growth of human colon, lung, and liver cancer cells [38,39]. Wheat gluten hydrolysates show antioxidant, antihypertensive, and immunomodulatory effects [40]. The chickpea and pumpkin seed protein hydrolysates showed antioxidant, hypocholesterolemic, Angiotensin Converting Enzyme (ACE)-inhibitory properties, metal-chelating ability, antihyperlipidemic, antitumor, and antiproliferative effects [41,42]. Therefore, plant-derived peptides have enormous potential as ingredients in developing healthcare and functional products. In Table 1 and Table 2, many specific peptides with specific bioactivities are listed, but they should not be considered the only ones with the activities.

### 2.3. Microbial Bioactive Peptides

Microbial fermentation is an alternative approach to produce bioactive peptides using bacterial enzymes. Many bacteria are used as starter cultures in industrial settings to produce strong proteolytic enzymes [49]. Thus, both primer and non-primer bacteria used in fermented foods have the potential to produce bioactive peptides. *Lactobacillales*, a diverse group of beneficial bacteria found in nature and the human gastrointestinal tract, are used to produce bioactive peptides. In addition to their physiological effects, these microorganisms contribute to fermented products’ texture and flavor with technological significance [5].

Numerous starter cultures utilized in the dairy industry possess a remarkable ability to degrade proteins. As a result, both starter and non-starter bacteria engaged in the fermentation process of dairy products can generate bioactive peptides. *Lactococcus lactis*, *L. helveticus*, and *L. delbrueckii* ssp. *bulgaricus* have been thoroughly investigated for their proficiency in protein hydrolysis [50]. However, all the bioactive functions mentioned in Table 1 and Table 2 are obtained by chemical analyses or a combination of chemical and in vitro biological analyses, and none of them are analyzed using animal models. In vitro studies may overlook issues like poor absorption and degradation of peptides into individual amino acids, making animal studies and human clinical trials essential. However, animal trials require significant investment and standardization of protocols to overcome challenges and should obtain health claims for functional food products in countries with regulations [51].

## 3. Bioactive Peptides in the Viability and Diversity of Gut Microbiota and Their Consequences to Human Health

**Figure 1:**
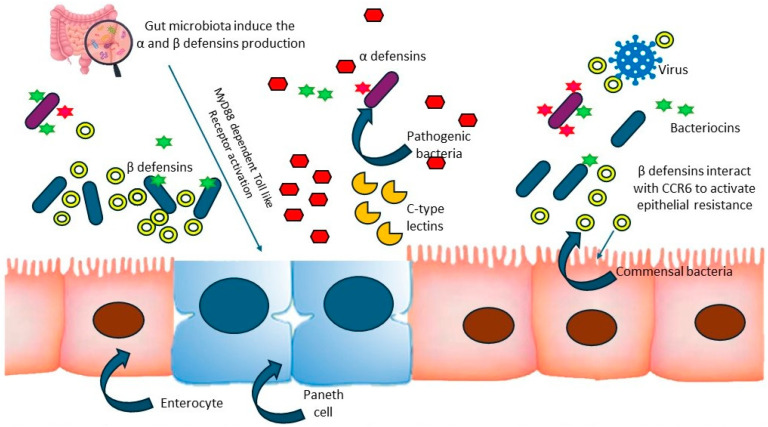
The mechanism of bioactive peptides on external bacteria and viruses. The release of bioactive peptides from intestinal cells and gut microbes inhibits the growth of external bacteria and viruses, maintaining the gut microbiota’s balance and stability. The gut microbiota is crucial for inducing the expression of antimicrobial peptides that defend against pathogens. These peptides are vital components of innate immunity, controlling pathogen growth within the intestine. Paneth cells can directly sense gut microbiota through MyD88-dependent TLR activation, leading to the expression of α-defensins and C-type lectins. β-defensin interacts with intestinal epithelial cells via CCR6, promoting epithelial resistance.

Naturally occurring or food protein-derived peptides with bioactivities have multiple functions in various biological processes, especially for promoting the health and function of the gut [11]. Previous literature reported that some bioactive peptides are nonspecific and might show multiple properties. However, only the major functions of peptides were studied without studying the influence of other peptides [52]. Also, it is very important to point out that peptides, including bioactive peptides, can hardly be absorbed in the gut and moved into the circulatory system because they should be hydrolyzed into mono-amino acids before penetrating into the circulatory system. Even if the microvilli can absorb some bioactive peptides, their amount would be small, and it is difficult to reach the critical levels to exert their positive functions [53]. Therefore, the dietary bioactive peptides could have functional effects on human health through their indirect effects on gut microbiota, such as maintaining a healthy gastrointestinal system by influencing barrier function, immune responses within the gastrointestinal system, and gut microbiota composition. The liberation of bioactive peptides from food proteins and gut microbes can impede the proliferation of external bacteria and viruses, thereby upholding the equilibrium and steadiness of the gut microbiota [53]. The mechanism through which bioactive peptides exert their effects on external bacteria and viruses is demonstrated in Figure 1.

### 3.1. Prebiotics and Probiotics

Prebiotics are food ingredients that the host cannot digest but selectively stimulate the growth of beneficial microbes in the host, thereby promoting the proliferation of probiotics. Probiotics are living microorganisms that offer health benefits to the host when consumed in various ways, such as supplements, fermented foods, probiotic-enriched beverages, and dairy products, with the required amount crucial for achieving the desired positive effects on health [54]. The effectiveness of probiotics is dose dependent, and the most frequently utilized probiotic genera are *Lactobacillus* and *Bifidobacterium* [55]. Probiotics help maintain a healthy balance of intestinal microorganisms and have a variety of beneficial bioactivities [56]. The primary source of nutrients for the growth of intestinal microorganisms is derived from undigested food components that reach the colon. These components include oligosaccharides, dietary fibers, undigested proteins, and endogenous sources. Any food that enters the colon, such as nondigestible carbohydrates, certain peptides, proteins, and some lipids, has the potential to act as a prebiotic. Oligosaccharides, such as lactulose, galactooligosaccharides, oligofructose, and maltooligosaccharides, are the most effective prebiotics studied thus far [57]. However, the methods for producing oligosaccharides are low in yield and expensive and only produce a single type of prebiotic [58,59,60]. Thus, identifying new types of prebiotics is of great interest to researchers. Recent research has demonstrated that proteins, protein hydrolysates, and peptides can stimulate the proliferation of probiotics [61].

Although some peptides have been identified as prebiotics, the interaction mechanisms between peptides and gut microbiota are poorly understood. Some scientists believe peptides in the gut act as a nitrogesn source and promote bacterial growth, while others believe they can improve polypeptide transport to precise locations in cells by acting as carriers or facilitators [61,62]. Raveschot et al. [63] reported that the growth of *Lactobacillus lactis* is heavily reliant on oligopeptides as a nitrogen source, and oligopeptides have been identified as the primary nitrogen source for lactic acid bacteria (LAB).

The activity of proteolytic enzymes influences the efficiency of protein utilization by bacteria [64]. Probiotics display varying abilities of using proteins in different conditions and do not require additional proteins when sufficient nutrients are available [65]. Prebiotics can substitute or supplement for probiotics. However, different types of prebiotics can stimulate the growth of specific types of gut bacteria [57]. Prebiotics have significant potential for altering the gut microbiota, but the changes are specific to individual strains and species and are difficult to predict in advance.

Conversely, probiotic bacteria can generate organic acids through the process of fermentation. When the organic acid concentrations reach a certain threshold, the bacteria may undergo autolysis, resulting in a decline in viable bacteria. Protein hydrolysates could be utilized to counteract and enhance the survival of the probiotic bacteria and preserve their numbers even in acidic environments [61].

### 3.2. Anti-Inflammatory Effects

Inflammation is a natural bodily response to infections, irritations, and injuries, resulting in the emergence of persistent illnesses such as cancer, obesity, asthma, and diabetes [66]. During inflammation, pro-inflammatory molecules, like tumor necrosis factor-α (TNF-α), interleukin (IL)-1, IL-6, IL-8, and interferon-g (INF-γ), are produced to protect the body. However, excessive production of pro-inflammatory molecules can cause damage to the tissues and impair immune functions [67]. The inflammatory response can be acute or chronic, and chronic inflammation has been linked to type 2 diabetes, inflammatory bowel disease (IBD), arthritis, atherosclerosis, and other cardiovascular diseases. Different cell types can interact with pro-inflammatory molecules, producing an amplified inflammatory response [68].

Peptides derived from various sources are capable of counteracting the effects of inflammation in cell culture studies. Dipeptides derived from yeast extracts and edible beans have anti-inflammatory activity in Caco-2 cells and specifically inhibit the secretion of pro-inflammatory cytokines IL-8, IL-6, and IL-1b while increasing the expression of anti-inflammatory cytokine IL-10 in the TNF-α-induced cells [69]. Tripeptides such as IPP (Ile-Pro-Pro) and VPP (Val-Pro-Pro) derived from the casein in milk exhibit anti-inflammatory characteristics [70]. Oligopeptides and polypeptides from various sources with anti-inflammatory properties inhibit the activity of pro-inflammatory cytokines and enzymes, thereby reducing inflammation [71,72]. However, these bioactivities have been examined using cell cultures and in vitro methods, and their health effects are not proven in in vivo systems. Therefore, studies using animal models and human subjects are needed to further explore the potential use of these bioactive peptides.

### 3.3. Antimicrobial Effects

Antimicrobial peptides (AMPs) regulate the interactions between commensal microbes and host tissues. Antimicrobial peptides exhibit antimicrobial effects via various mechanisms, including sequestering essential growth nutrients and disrupting bacterial membranes [61]. These peptides, also known as host defense peptides, act as an important barrier against pathogenic bacterial invasion of the body [73]. Antimicrobial peptides are classified into four categories based on their origin: microbial, animal, plant, and others, and their antibacterial effects vary depending on their sources [74]. Antimicrobial peptide functions are in three stages: the initial attraction to the bacterial cell wall, subsequent binding to the cell membrane, and eventual insertion of peptides into the host cell membrane, causing membrane permeabilization. Antimicrobial peptides can use various antimicrobial strategies, including membrane destabilization, bacterial cell filamentation by peptide insertion, inhibiting DNA replication, and hindering the membrane proteins involved in septum formation. Antimicrobial peptides also have lectin-like properties and anti-toxin activities [75].

Milk is a good source of nutrients and has a variety of biological functions that are primarily attributed to its peptides and proteins. Milk’s antimicrobial properties come mainly from lactoferrin, lactoperoxidase, and lysozyme. Milk also contains antibacterial peptides, crucial parts of innate immunity, and helps protect animals against possible harmful microorganisms [76]. Milk proteins nourish and protect newborns and produce bioactive peptides that can protect against infectious agents. The peptides that can be obtained from lactoferrin, caseins (αS1, αS2, β, and κ-casein), α-lactalbumin, β-lactoglobulin, protease-peptone-3, and lysozyme can serve as antibacterial agents in the fields of therapy, functional foods, and infant formulas [77]. Microorganisms such as bacteria and fungi are a source of antimicrobial peptides. Nisin and gramicidin are commonly recognized peptides derived from *Lactococcus lactis*, *Bacillus subtilis*, and *Bacillus brevis* [78]. Epithelial cells can also produce antimicrobial peptides which help protect the animal body from harmful microorganisms. The primary sources of antimicrobial peptides in the gastrointestinal tract are Paneth cells and enterocytes, although immune cells in the tissue can also produce these molecules [79]. Defensins, cathelicidins, and regenerating Gene III alpha/beta/gamma are the three primary types of antimicrobial peptides in the gut. These peptides have dual roles: preventing pathogen infection and influencing the microbiome composition. Their antimicrobial activity is broad spectrum and can interfere with microbial growth and metabolism [80]. They can also affect immune responses and cell signaling that impact innate and adaptive immunity. Antimicrobial peptides could be a therapeutic approach to treat diseases associated with microbiome imbalances, especially in light of the growing problem of antibiotic-resistant pathogens [81].

### 3.4. Anti-Obesity Effects

Previous research suggested that gut microorganisms could help people lose weight by regulating their energy balance and food consumption. Furthermore, they can reduce inflammation caused by obesity by modifying the expression of inflammation-related transcription factors. Protein hydrolysates and peptides can inhibit the growth of gut bacteria that cause obesity. These compounds promote the growth of beneficial gut bacteria while improving their resistance to extreme pH conditions. Hence, protein hydrolysates/peptides can be considered promising therapeutic agents for obesity and the associated complications, enabling the development of innovative strategies to combat obesity [82].

The gut’s microbial makeup can vary depending on acidity and oxygen levels. *Firmicutes*, *Lactobacilli*, and *Proteobacteria* mostly populate the area closer to the beginning of the digestive tract, while the region further down is mainly inhabited by *Bacteroidetes*, *Firmicutes*, and *Akkermansia muciniphila* [83]. Recent research [84] indicated a noticeable shift in the makeup and structure of the gut microbiota in individuals who are obese. Specifically, obese individuals tend to have a higher prevalence of *Firmicutes* clusters, while those with lean body mass tend to have more *Bacteroidetes*. The consensus among studies is that this gut microbiota imbalance can disrupt the host’s capacity to extract energy from food efficiently and consequently influence energy storage and utilization in adipose tissues, ultimately contributing to changes in body weight [82].

Obesity is commonly linked to dietary habits as they can influence the makeup of the gut microbiota. Consequently, recent attempts to address obesity have centered around the role of gut microbiota in weight gain. Certain types of gut microbiota can increase the likelihood of obesity by affecting the body’s physiological pathways involved in weight gain [85]. Requena et al. reported changes in gut microbiota composition when egg white hydrolysate peptides were fed to obese rats [20]. Han et al. showed that pepsin-hydrolyzed peptides from soybean 7S globulin lowered body weight and influenced gut microbiota composition in obese individuals [86]. In rats, feeding with hydrolyzed α-lactalbumin derived from cows increased the proportion of Bacteroidetes/Firmicutes and a greater relative abundance of Lachnospiraceae and Blautia in the gut microbiota [87]. Collagen peptides derived from the skin of walleye pollock reduced obesity by influencing the gut microbiota in mice fed a high-fat diet. The mice that were given collagen peptides had an increased number of beneficial bacteria, such as *Lactobacillus*, *Akkermansia muciniphila*, *Parabacteroides*, and *Odoribacter* spp., and fewer bacteria, such as *Erysipelatoclostridium* and *Alistipes*, that cause inflammation in the gut [88,89]. The pH balance of the gut environment fluctuates between acidic and alkaline states, and fermentation by gut bacteria produces organic acids, which can alter the pH balance. Some bacteria are sensitive to pH changes, and exposure to highly acidic conditions can cause autolysis, decreasing the number of viable colonies in the gut [61]. The findings confirm that consuming hydrolysates/peptides can help sustain a healthier balance of gut microbiota and decrease obesity-associated effects on the host, which implies that hydrolysates/peptides can act as prebiotic agents for managing inflammation and oxidative stress triggered by obesity-induced dysbiosis in the gut microbiota of obese people.

### 3.5. Modulating Gut–Brain Axis and Enhancing Gut Immunity

The communication between the gastrointestinal tract (GIT) and the brain is a multifaceted and reciprocal process that occurs through the circulation of blood and the cranial nerves. Enteroendocrine cells (EECs) release hormones that stimulate the brain through chemical signals in the bloodstream or neural pathways [90]. The enteric nervous system (ENS) is sometimes referred to as the “second brain” because it contains millions of neurons capable of controlling many aspects of gut function, such as regulating digestive enzyme secretion, controlling blood flow to the gut, and coordinating smooth muscle contraction in the digestive tract [91]. The enteric nervous system (ENS) interacts with the central nervous system (CNS) through various channels, including the vagus nerve, the primary conduit for transmitting sensory and motor signals between the gut and the brain. The vagus nerve connects the brainstem to the ENS and innervates many organs in the body, including the heart, lungs, liver, pancreas, and gut [92]. Numerous scientific investigations have proposed that modifying one’s everyday diet may benefit the microbiota–gut–brain axis, potentially preventing neurodegenerative diseases. Maintaining a healthy gut and a balanced gut microbiota composition is closely linked to diet composition and dietary practices. An imbalance in the gut microbiota, also known as dysbiosis, can disrupt the communication between the gut and the brain in both directions [93].

Peptides derived from dietary protein digestion or consumed as supplements play a crucial role in regulating gut microbiota and maintaining the balance of reactive oxygen species (ROS) in the gut, which is important for overall gut and host health [94]. Furthermore, certain peptides have therapeutic benefits for neurodegenerative illnesses. The gut–brain axis plays a significant role in the onset and advancement of various neurological conditions, such as Alzheimer’s disease (AD), Parkinson’s disease (PD), and autism [95].

The peptides derived from fermented dairy products show neuroprotective effects and alleviated symptoms associated with Alzheimer’s disease and other neurodegenerative conditions [96]. Also, those peptides ameliorate cognitive impairment by suppressing the activity of monoamine oxidase-B, an enzyme responsible for breaking down neurotransmitters such as dopamine. Inhibiting monoamine oxidase-B increases dopamine levels in brain tissue, improves cognitive function, and slows neurodegenerative disease progression [97]. These peptides reduce inflammation and oxidative stress and promote nerve cell survival in the brain. The Maillard reaction products of soybean peptides reduce oxidative stress and systemic inflammation that directly contributes to cognitive decline and aging [61]. The digestion products of whey and casein proteins stimulate the secretion of cholecystokinin (CCK) and glucagon-like peptide-1 (GLP-1) hormones, which are associated with satiety and food control [98]. These bioactive peptides improve gut microbiota and intestinal mucosa immunity, suggesting potential interventions to impact the gut–microbiome–immune axis positively. Bao and Wu [11] reported that dietary peptides are involved in various aspects of intestinal mucosal functions, including cell proliferation, microbial diversity, mucin production, tight junction protein expression, and antioxidant and immune cell activities, and are crucial in regulating gastrointestinal barrier functions that protect against pathogens and inflammation and promote overall gut health.

## 4. Assessing the Safety and Potential Risks of Peptides in Gut Microbiota

Bioactive peptides can exhibit diverse biological functions and involve numerous physiological processes that benefit human health. However, the safety of bioactive peptides is also an important consideration for their use in clinical studies and food applications. Thus, it is critical to ensure the safety of peptides despite the common misconception that bioactive peptides are safe because they are derived from food proteins using food-grade proteases. It is critical to acknowledge the existence of many naturally occurring toxic proteins and peptides [99]. Consuming peptides from food or concentrated forms is believed to have no adverse physiological effects [4]. The potential acute and repeated oral toxicity of casein hydrolysate that contains antihypertensive peptides RYLGY and AYFYPEL showed that a maximum oral dose in the 4-week repeated dose had non-observable toxic effects. Furthermore, no toxicity was observed after a single oral-limit dose, indicating a low potential for oral toxicity [100].

However, many naturally occurring peptides and enzymes in plants, animal by-products, and dried food products can potentially be toxic to unicellular and multicellular organisms [101,102]. Peptide toxicity is the underlying cause of celiac disease. Phallotoxins and amatoxins are two toxic peptides present in mushrooms, and those two cause severe damage to the liver by blocking RNA polymerase II activity, leading to impaired protein synthesis, cellular death, acute liver failure, and ultimately mortality [103]. Auestad and Layman [104] identified specific milk peptides that can cause allergies. These peptides are derived from casein and whey and serve various functions, including ACE inhibition.

## 5. The Implications of Research on Bioactive Peptides and Gut Microbiota

### 5.1. Development of New Therapeutics Using Bioactive Peptides

Exploration of bioactive peptides has emerged as a pivotal avenue for advancing novel therapeutic strategies due to their distinct biological activities [105]. Researchers aim to create innovative treatments that address various medical challenges by harnessing the inherent properties of biopeptides. The ACE-inhibitory properties of bioactive peptides in living organisms were studied through systematic blood pressure measurements in spontaneously hypertensive rats (SHRs) within in vivo settings. The assessments used diverse administration routes, including intravenous, intraperitoneal injections, and oral gavage, to determine the impact of these peptides on blood pressure levels. Bioactive peptides from food sources were suggested to offer a natural approach to controlling blood pressure without some side effects associated with traditional ACE inhibitors. These bioactive peptides work by inhibiting the enzyme that causes blood vessels to constrict, resulting in reduced blood pressure [106]. Chen et al. [107] reported that peptides could be passed through the intestinal membrane by active transport, passive transcellular diffusion, transcytosis, and tight junctions. However, absorbing the intact lactotripeptide in biologically functional amounts through the intestinal membrane and penetration into the bloodstream would be marginal. It is well accepted that single amino acids and di- and tripeptides can pass through the brush border membrane, but almost all the di- and tripeptides are digested into single amino acids by various digestive enzymes before penetrating into the bloodstream [108]. Even if some small peptides pass through the brush border and into the bloodstream, their amount should be very low. Therefore, it is almost impossible for the functional peptides to move to the target organs and generate their expected functions [109,110].

Bioactive peptides with antimicrobial properties offer a remarkable breadth of activity, capable of eliminating bacteria, fungi, viruses, and even cancer cells. Their potency is underpinned by a unique mode of action that challenges bacteria to develop resistance [111]. The significance of these peptides extends further as they possess the exceptional ability to hinder the formation of biofilms—an intricate alliance of microorganisms that cling to surfaces and evade immune responses and conventional antibiotics. These biofilms contribute to persistent infections linked to medical devices and pose formidable challenges to eradication. Remarkably, some antimicrobial peptides exhibit the power to impede biofilm formation or dismantle established biofilm fields [112]. In addition to their formidable antimicrobial prowess, antimicrobial peptides exhibit a virtue of paramount importance: their minimal toxicity towards mammalian cells. This trait positions them as a prospective therapeutic arsenal against infections, capable of effectively combating pathogens while protecting the host from substantial harm. However, employing antimicrobial peptides as clinical antimicrobial agents is still nascent, and addressing challenges between their present promise and integration into established clinical practices is important [113].

Notably, casein and phosvitin phosphopeptides possess multifaceted benefits. Their mineral-binding attributes are crucial in facilitating the absorption of vital minerals like calcium, iron, and magnesium [114,115]. As convergence of science and health, the potential of casein and phosvitin phosphopeptides to enhance therapeutic strategies emerges as a remarkable avenue of exploration. Many studies proposed their anticancer potential attributed to shielding cells against the pernicious onslaught of reactive oxygen species (ROS), including hydrogen peroxide, superoxide anion, and hydroxyl radicals. While such shielding proves effective in cell culture, its translation to animal models remains challenging [116]. The journey of formulating novel therapies hinging on these revelations holds tantalizing prospects in the combat against cancer and the refinement of cancer-related interventions in the days ahead.

Similarly, some peptides harbor the ability to forestall damages orchestrated by reactive nitrogen species (RNS), encompassing peroxynitrite and nitric oxide, the latter recognized as the most potent oxidant [117]. It is imperative to note that ROS/RNS have garnered infamy as culprits behind a gamut of chronic ailments, spanning diabetes, renal impairment, Parkinson’s disease, cardiovascular events, and an array of inflammatory disorders, skin aging, cancers, cardiovascular maladies, osteoporosis, and gastrointestinal afflictions [118,119]. The vista that unfolds before us beckons toward the horizon of bioactive peptides as therapeutic forerunners. However, to fully harness their potential, the roadmaps of ongoing research necessitate robust expeditions of intense pre-clinical evaluations, and clinical trials will etch their safety and efficacy onto the annals of specific medical interventions. As the curtain lifts on the future of medicine, bioactive peptides stand poised to play a transformational role, offer solutions to a diverse array of ailments, and elevate human health to new heights.

### 5.2. The Use of Bioactive Peptides in Food Processing

Bioactive peptides have gained significant attention as functional food ingredients due to their potential health benefits. Bioactive peptides can be incorporated into food products and improve food quality by influencing physical and chemical properties [4]. Furthermore, bioactive peptides can help change the viscosity of food, affecting its mouthfeel and texture [120]. Bioactive peptides derived from food by-products, such as collagen, whey, and soy, have emulsifying properties due to their amphiphilic nature. Plant-derived peptides, such as those from potatoes [121] and soybeans [122], have been identified as potential emulsifiers. These peptides are extracted from food by-products or produced via enzymatic hydrolysis and used as natural emulsifying agents in food and cosmetic products [123].

Antioxidant peptides can be used in food products to prevent lipid oxidation and extend shelf life. They also provide health benefits by protecting against oxidative stress and lowering the risk of chronic diseases. Farvin et al. [124] discovered fish protein hydrolysates containing antioxidant peptides that prevent lipid oxidation in fish oil emulsions. Antimicrobial peptides can be used in food products as natural preservatives to inhibit the growth of spoilage and pathogenic microorganisms. Pan et al. [125] reported that whey protein hydrolysates containing antimicrobial peptides inhibit the growth of common foodborne pathogens. However, emphasizing specific peptides as antioxidant or antimicrobial agents in food processing should be avoided because it is not practical to differentiate them according to their specific function. These peptides have gained attention in the food industry due to their potential health benefits and functional properties.

## 6. Future Research on the Bioactive Peptides and Gut Microbiota and Their Applications

Human gut microbiota comprises various microorganisms, including bacteria, fungi, and archaea. Bacteroidetes and Firmicutes phyla are the most prevalent among bacterial species, while the rest of the population belongs to *Actinobacteria*, *Proteobacteria*, *Fusobacteria*, *Spirochaetes*, *Verrucomicrobia*, and *Lentisphaerae*. These microorganisms significantly outnumber human cells and genes in the gut, and they play essential roles in various health functions of an individual. The gut microbiota is associated with maintaining homeostasis, regulating energy balance, modulating allergies, controlling appetite, and contributing to various diseases such as cardiovascular diseases, immunity, obesity, and diabetes. The microorganisms present in the colon play a crucial role in facilitating the digestion and absorption of nutrients, breaking down indigestible dietary components, and supplying micronutrients to the host. Some groups of colonic bacteria, such as *Lactobacilli* and *Bifidobacteria*, possess immunomodulatory properties, produce digestive enzymes, and help restore the gut microbiome’s equilibrium following antibiotic treatment. The complex interactions within the gut microbiota highlight the importance of studying gut microorganisms for maintaining human health. Several factors can affect the composition of gut microbiota, with diet being the most significant. Bioactive peptides can have a prebiotic effect by selectively promoting the growth of beneficial bacteria in the gut. They can also have an antimicrobial effect by inhibiting the growth of harmful bacteria.

Moreover, bioactive peptides possess anti-inflammatory characteristics, which may assist in diminishing gut inflammation and promoting healthy gut microbiota. Overall, bioactive peptides have the potential to modulate the gut microbiota and promote gut health. Therefore, bioactive peptides can be considered gut microbiota modulators. While di- and tripeptides are known to be absorbed by the intestine, only limited information on the absorption of higher-molecular-weight bioactive peptides is available [109]. After absorption, these bioactive peptides are carried by the bloodstream and interact with specific receptors, or the concentrations reaching the target cells or receptors are measurable for sustained responses [126]. The study of bioactive peptides is complex, and more research is required to understand their fate in the human body fully. Specific factors such as the nature of the peptide and its chain length influence absorption. Also, the extent of absorption might vary for each bioactive peptide [127].

## 7. Conclusions

Bioactive peptides have positive effects on gut microbiota. Bioactive peptides help maintain a balanced microbial environment, counteract obesity, and enhance gut–brain communication and immunity. Bioactive peptides play a significant role in modulating the immune response, particularly inflammation and related diseases. These peptides possess immunomodulatory properties, interacting with immune cells and signaling pathways and producing specific immune mediators. Some bioactive peptides have direct and indirect anti-inflammatory effects and reduce pro-inflammatory cytokines, thus helping manage chronic inflammatory conditions. Moreover, bioactive peptides influence gut health and gut microbiota, impacting the immune system indirectly. A balanced gut microbiota supports a well-regulated immune response, and the strengthened gut immunity offers protection against infections, which reduces inflammation and supports overall gut health. These peptides, with their diverse functionalities, encompass prebiotic and probiotic effects. By selectively stimulating the growth of beneficial bacteria and inhibiting pathogens, they contribute to gut health and immune function.

Consequently, bioactive peptides are crucial in maintaining a well-functioning gastrointestinal system. They promote a balanced gut microbiota and reinforce gut immunity, which makes them valuable in managing inflammation and associated diseases. Further research in this field on the mechanisms of how dietary bioactive peptides can influence hosts should be elucidated. This knowledge will help optimize the design of functional foods and dietary interventions to target specific health benefits related to gut health and beyond. While there is promising evidence for the potential health benefits of bioactive peptides, research to establish their bioavailability, absorption, interaction with gut microbiota, and efficacy in human subjects is needed. Understanding these factors is essential for harnessing the full potential of bioactive peptides and incorporating them into functional foods, therapeutics, and nutraceuticals that can effectively promote human health.

## Figures and Tables

**Table 1:** Animal-derived bioactive peptides.

Animal Source	Main Protein	Type of Peptides Derived	Bioactivity	Analysis Method	Reference
Milk	Casein	Casomorphins	ACE-inhibitory, antimicrobial	Biochemical, Microbial	[26,27]
	Whey protein	α-Lactorphin	ACE-inhibitory, antioxidant	Chemical	[28]
Fish	Tuna	QGD, GEQSN, PKK, GPQ, GEEGD	Antioxidant	Chemical	[29,30]
Shellfish	Oyster	Oyster peptide hydrolysate	Antioxidant, anti-inflammatory	Chemical	[31]
	Shrimp	Val-Gly-Pro, Isoleu-Pro-Pro	ACE-inhibitory, antimicrobial	Enzymatic	[32]
Meat	Beef	GFHI, DFHING, FHG, GLSDGEWQ	Antioxidant, anti-obesity	Chemical, Enzymatic	[33]
	Pork	The titin-derived pentapeptides KAPVA, PTPVP	ACE-inhibitory, anti-inflammatory	Chemical, Enzymatic	[34]
	Chicken	LKA, LKP, LAP, IKW, FQKPKR, FKGRYYP, IVGRPRHQG	ACE-inhibitory	Enzymatic	[35]
	Duck	Trp-Tyr-Pro-Ala-Pro	ACE-inhibitory, antioxidant	Chemical, Enzymatic	[36]
Insects	Cricket	Defensin-1	antimicrobial	Microbial	[24]
	Silkworm	SPKFCW, DQDPFRP, PDPSKF	Antimicrobial	Microbial	[25]

**Table 2:** Plant-derived bioactive peptides.

Plant Source	Peptide Producing Compound	Type of Peptides	Health Benefits	Reference
Soybeans	Lunasin	SKWQHQQDSCRKQKQ, GVNLTPCEKHIMEKIQ, GRGDDDDDDDDD	Anti-inflammatory, anticancer, cholesterol-lowering activity	[43]
Wheat	Gluten exorphins	GE A5, GE C5	Opioid-like activity	[44]
Rice	Rice bran peptides	Trypsin-hydrolyzed rice bran	Antioxidant, anti-inflammatory, immune-modulating activity	[45]
Peas	ACE-inhibitory peptides	KEDDEEEEQGEEE	Blood pressure regulation, cardiovascular health activity	[46]
Chickpeas	ACE-inhibitory and antioxidant peptides	Leu-Thr-Glu-IIe-IIe-Pro	Blood pressure regulation, cardiovascular health, cellular protection, oxidative stress reduction	[47]
Hemp seeds	Antioxidant and anti-inflammatory peptides	WVSPLAGRT (H2), IGFLIIWV (H3)	Cellular protection, oxidative stress reduction, inflammation reduction	[48]

## Data Availability

No new data were created or analyzed in this study. Data sharing is not applicable to this article.
